# Urinary Polyamine Biomarker Panels with Machine-Learning Differentiated Colorectal Cancers, Benign Disease, and Healthy Controls

**DOI:** 10.3390/ijms19030756

**Published:** 2018-03-07

**Authors:** Tetsushi Nakajima, Kenji Katsumata, Hiroshi Kuwabara, Ryoko Soya, Masanobu Enomoto, Tetsuo Ishizaki, Akihiko Tsuchida, Masayo Mori, Kana Hiwatari, Tomoyoshi Soga, Masaru Tomita, Masahiro Sugimoto

**Affiliations:** 1 Department of Gastrointestinal and Pediatric Surgery, Tokyo Medical University, 6-7-1 Nishishinjuku, Shinjuku, Tokyo 160-0023, Japan; nakajimatetsushi55@hotmail.com (T.N.); k-katsu@tokyo-med.ac.jp (K.K.); hiroshi.kuwabara.bob@gmail.com (H.K.); rsoya@tokyo-med.ac.jp (R.S.); menomoto@yahoo.co.jp (M.E.); wbc15000@yahoo.co.jp (T.I.); akibobo@hotmail.com (A.T.); 2 Institute for Advanced Biosciences, Keio University, Tsuruoka, Yamagata 997-0052, Japan; morimasa@ttck.keio.ac.jp (M.M.); cana143@ttck.keio.ac.jp (K.H.); soga@sfc.keio.ac.jp (T.S.); mt@sfc.keio.ac.jp (M.T.); 3 Research and Development Center for Minimally Invasive Therapies Health Promotion and Preemptive Medicine, Tokyo Medical University, Shinjuku, Tokyo 160-8402, Japan

**Keywords:** colorectal cancer, polyamine, urine, liquid chromatography-mass spectrometry, machine learning

## Abstract

Colorectal cancer (CRC) is one of the most daunting diseases due to its increasing worldwide prevalence, which requires imperative development of minimally or non-invasive screening tests. Urinary polyamines have been reported as potential markers to detect CRC, and an accurate pattern recognition to differentiate CRC with early stage cases from healthy controls are needed. Here, we utilized liquid chromatography triple quadrupole mass spectrometry to profile seven kinds of polyamines, such as spermine and spermidine with their acetylated forms. Urinary samples from 201 CRCs and 31 non-CRCs revealed the *N*_1_,*N*_12_-diacetylspermine showing the highest area under the receiver operating characteristic curve (AUC), 0.794 (the 95% confidence interval (CI): 0.704–0.885, *p* < 0.0001), to differentiate CRC from the benign and healthy controls. Overall, 59 samples were analyzed to evaluate the reproducibility of quantified concentrations, acquired by collecting three times on three days each from each healthy control. We confirmed the stability of the observed quantified values. A machine learning method using combinations of polyamines showed a higher AUC value of 0.961 (95% CI: 0.937–0.984, *p* < 0.0001). Computational validations confirmed the generalization ability of the models. Taken together, polyamines and a machine-learning method showed potential as a screening tool of CRC.

## 1. Introduction

Colorectal cancer (CRC) is the second and third most frequently diagnosed cancer among males and females, respectively, both in the USA [1] and worldwide in 2012. In Japan, the incidence of CRC has significantly increased, and this country is regarded as having one of the highest incidences [2,3,4]. Tumor markers—such as serum carcinoembryonic antigen (CEA), CA19-9, and CA15-3—have been used to identify patients with CRC, but more accurate screening markers need to be developed. The development of screening biomarkers with higher sensitivity and specificity are still necessary.

The naturally-occurring polyamines, spermidine and spermine, and their precursors, diamine and putrescine, are aliphatic polycations which are ubiquitously observed in mammalian cells. Their essential role in the proliferation and differentiation of prokaryotic and normal eukaryotic cells is well established [1]. Polyamines, such as spermidine and spermine, are produced in almost all cells but are particularly highly produced in rapidly growing cells. Arginine, one of the amino acids, is converted to ornithine by arginase (EC 3.5.3.1), and ornithine is catalyzed by ornithine decarboxylase (ODC) (EC 4.1.1.17) to produce putrescine, which is a precursor metabolite of polyamines. Spermidine and spermine are synthesized from putrescine and decarboxylated *S*-adenosylmethionine [5]. These polyamine metabolites constitute loops through spermine/spermidine *N*_1_-acetyltransferase (SSAT), *N*_1_-acetylpolyamine oxidase (APAO), and spermine oxidase (SMO). These metabolize spermine to *N*_1_-acetylspermine, spermidine to *N*_1_-acetylspermidine, and spermine to spermidine, respectively [5].

**Figure 1:**
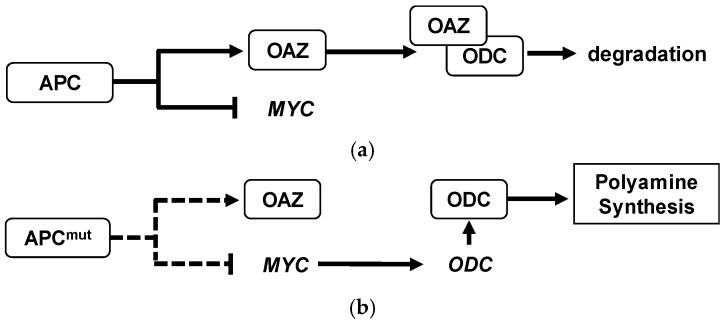
Adenomatous polyposis coli (APC) regulation of polyamine synthesis in colorectal cancer (CRC). (**a**) Normal cell. Tumor suppressor wide-type APC suppresses transcription of protooncogene *MYC* and also acts to regulate the ornithine decarboxylase (ODC) antizyme (OAZ) which degrades ODC. (**b**) Cancer cell. Mutated or deleted APC induces a decrease in OAZ and reduction of *MYC* suppression, which results in the increase of the expression of *ODC* gene. Consequently, the polyamine synthesis is activated in cancer cells. Dashed arrows indicate less effect compared to the solid arrows.

The enhanced activity of polyamine pathways in CRC is well known. For example, the first rate limiting enzyme, ODC, is negatively regulated by the adenomatous polyposis coli (APC) tumor-suppressor gene in colonic mucosal tissue [6]. The loss of APC function would activate ODC enzyme, resulting in the activation of polyamine biosynthesis [7]. The schematic diagram of APC on polyamine metabolisms was described (Figure 1). These metabolites were secreted from tumor tissue and spread to surrounding tissues and blood vessels [5]. Therefore, a combination of polyamine or metabolites have been used for the development of non- or low-invasive screening, such as blood, urine, and fecal-based tests [8,9], to identify patients with CRC or polyps, a precursor of CRC. Elevated concentrations of urinary *N*_1_,*N*_12_-acetylspermine of CRC has been consistently observed in various studies [10,11]. However, all reports claimed the change of a single polyamine is not enough to diagnose CRC, i.e., low specificity as a biomarker. Nowadays, we have various pattern recognition and machine learning algorithms, and the use of these method has the potential to show better accuracy.

The use of machine learning methods with metabolomics profiles in biofluid and tumor samples has been accumulated for diagnosis and screening purposes. For example, deep learning methods were used for estrogen receptor status in breast cancer tissue samples [12]. Orthogonal partial least squares discriminant analyses ranked the predictive metabolites and, subsequently, a decision tree was developed for discriminating bladder cancer using urinary metabolite profiles [13]. Particularly, partial least squares-discriminant analysis (PLS-DA) has been frequently used to select variables showing discriminant ability of given two-class problems, e.g., prediction of colon CRC progression using serum metabolites [14] and discrimination of CRC from non-CRC groups using metabolite profiles in serum [15] and urine samples [16]. These machine learning methods would contribute to enhance the discrimination ability by combining the predictive abilities of multiple metabolites. However, these methods are supervised and, therefore, various validations are key factors to prevent overfitting. Even in rigorous validation, the use of biologically reasonable metabolites is also important to eliminate optimistic prediction.

In this study, we utilized liquid chromatography-mass spectrometry (LC-MS) for simultaneous quantification of urinary polyamines. The discrimination ability of CRC from benign and healthy controls were assessed. There are many reports on the urinary metabolite profiles in various subjects. Therefore, diurnal and day-to-day differences were investigated to assess the variation of observed urinary metabolites and, subsequently, the discrimination abilities of single and multiple markers were evaluated. To enhance the discrimination ability of these markers, a machine-learning method was utilized.

## 2. Results

**Figure 2:**
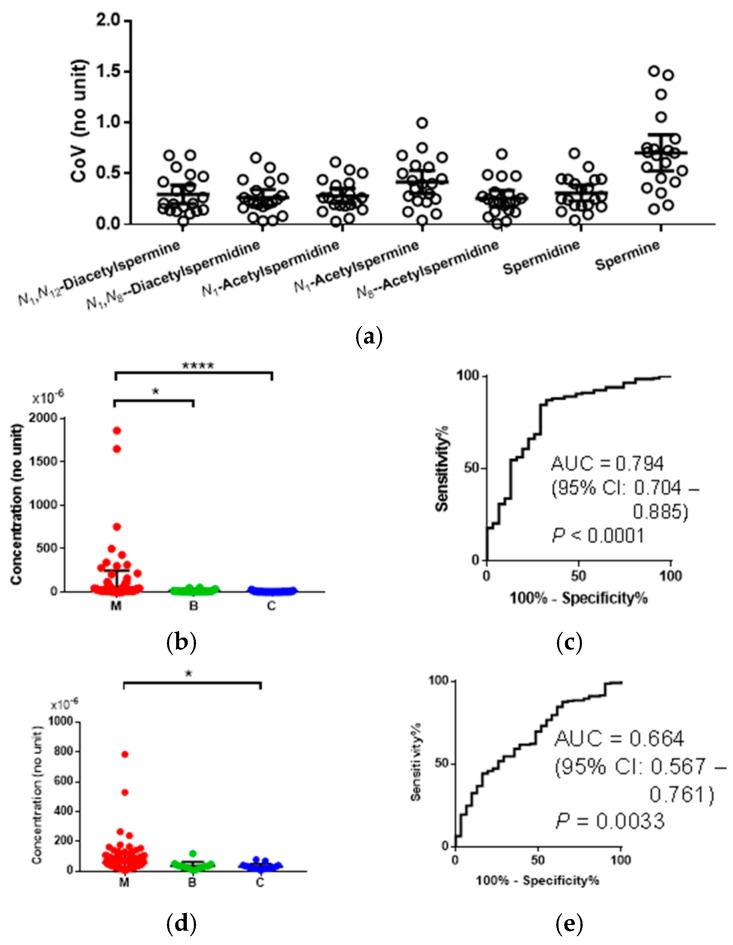
The producibility and discrimination abilities of polyamines. (**a**) The coefficient of variation (CoV) of healthy control subjects. Horizontal bars indicated means and 95% confidential intervals. Overall, 20 samples were used. Horizontal bars indicated the mean and 95% confidential interval. (**b**) *N*_1_,*N*_12_-acetylspermine concentration divided by creatinine one and (**c**) the ROC curve to discriminate M (*n* = 201) from B + C (*n* = 31). (**d**) *N*_1_,*N*_8_-acetylspermidine divided by creatinine one and (**e**) the ROC curve to discriminate M (*n* = 201) from B + C (*n* = 31). Horizontal bars indicated the mean and SD (**b**,**d**). Red, green, and blue indicated the data of M, B, and C, respectively. *p*-values were calculated using the Kruskal-Wallis test with Dunn’s post-test * *p* < 0.05 and **** *p* < 0.0001.

An overview of the observed data is summarized here. The subject information is summarized in Table 1. The quantified polyamines and their discrimination ability are depicted in Figure 2. The coefficient of variation (CoV) values of quantified polyamines in the urinary samples collected from controls (C) are depicted in Figure 2a. The data included all collected samples and, therefore, both diurnal and day-to-day variations were included. Of these, spermine showed the largest mean CoV (0.70) and the others showed 0.25–0.42. For example, the difference of the mean concentration of *N*_1_,*N*_12_-acetylspermine in malignant (M) (mean = 59.9 × 10^−6^ (no unit)) and C (mean = 7.93 × 10^−6^) was 52.0 × 10^−6^, which was larger than 23-fold of the standard deviation (SD) of the concentrations of C (Figure 2b). The difference of the mean concentration of *N*_1_,*N*_8_-acetylspermidine of M (57.9 × 10^−6^) and C (31.0 × 10^−6^) was 26.9 × 10^−6^, which was larger than 3.3-fold of the SD of the concentrations of C (Figure 2d). Among all polyamines, only *N*_1_,*N*_12_-acetylspermine and *N*_1_,*N*_8_-acetylspermidine showed a high area under the receiver operating characteristic (ROC) curves (AUC), allowing for discrimination of CRC from healthy controls; AUC = 0.794 (95% CI: 0.704–0.885, *p* < 0.0001) (Figure 2b,c) and AUC = 0.664 (95% CI: 0.560–0.755, *p* = 0.0022), respectively. The *N*_1_,*N*_12_-acetylspermine showed significantly elevated levels compared to both healthy controls and benign cases (Figure 2d,e). The differences between M and C were enough, even though the diurnal and day-to-day variation was considered.

**Figure 3:**
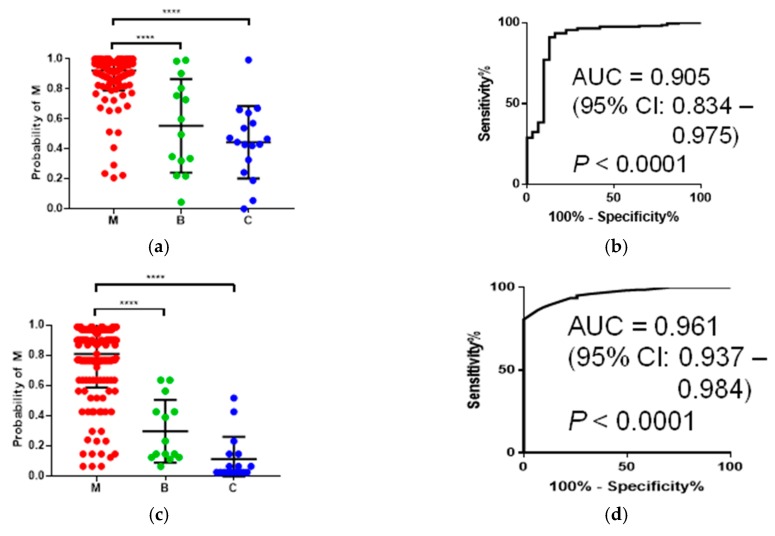

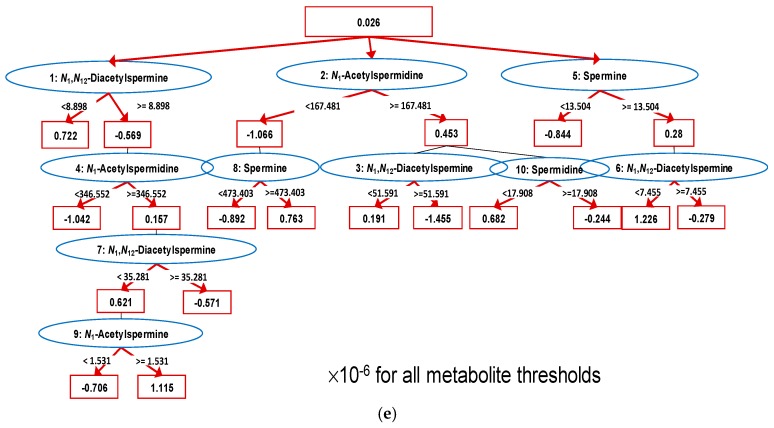
Discrimination abilities of mathematical models. (**a**) Distribution of predicted probabilities of malignancy (M) calculated by multiple logistic regression (MLR) and (**b**) its ROC curve. (**c**) Distribution of predicted probability of the ADTree model and (**d**) its ROC curve. (**e**) The ADTree model to discriminate M (*n* = 201) from B + C (*n* = 31). Total scores <0 and >0 indicated the higher and lower probability of M. All values of the thresholds in this tree should be ×10^−6^ to calculate the probability of M. *p*-values of (**a**) and (**c**) were calculated using the Kruskal–Wallis test with Dunn’s post-test. **** *p* < 0.0001.

To assess the discrimination ability of multiple polyamines, we developed a multiple logistic regression (MLR) model incorporating multiple polyamines. A stepwise feature selection procedure selected 6 and 2 as independent variables in the models to discriminate M from benign (B) + C and B from C + M, respectively (Table 2). The former model showed AUC = 0.905 (95% CI: 0.834–0.975, *p* < 0.0001), with a higher accuracy compared to *N*_1_,*N*_12_-acetylspermidine alone (Figure 3a,b). The latter model showed AUC = 0.763 (95% CI: 0.650–0.875, *p* = 0.001).

We utilized alternative decision tree (ADTree)-based machine-learning methods to enhance the discrimination ability of multiple polyamines. The boosting number (i.e., the number of nodes in a tree) was optimized based on cross validation (CV). The AUC values showed peaks (i.e., increased and subsequently decreased along with the boosting number), and the boosting number was determined at this peak. An ADTree model to discriminate M from B + C showed AUC = 0.961 (95% CI: 0.937–0.984, *p* < 0.0001) (Figure 3c,d). The model included 10 nodes optimized by CV procedures (Figure 3e). In contrast, the ADTree model with 12 nodes to discriminate B from M + C showed lower AUC values; the AUC was 0.763 (95% CI: 0650–0.875, *p* = 0.001). The bootstrap and CV analyses of the former model resulted in a median AUC = 0.989 (95% CI: 0.988–0.990) and AUC = 0.957 (95% CI: 0.955–0.958), respectively.

The discrimination abilities of tumor markers, *N*_1_,*N*_12_-acetylspermidine, and MLR and ADTree models for each stage are summarized in Table 3. Optimal cut offs were determined by ROC curves for *N*_1_,*N*_12_-acetylspermidine and MLR and ADTree models. All data showed significant differences based on the stage. The ADTree resulted in no false positives while MLR produced four false positives for all B and C subjects using the optimal cut-off calculated from ROC curves.

**Figure 4:**
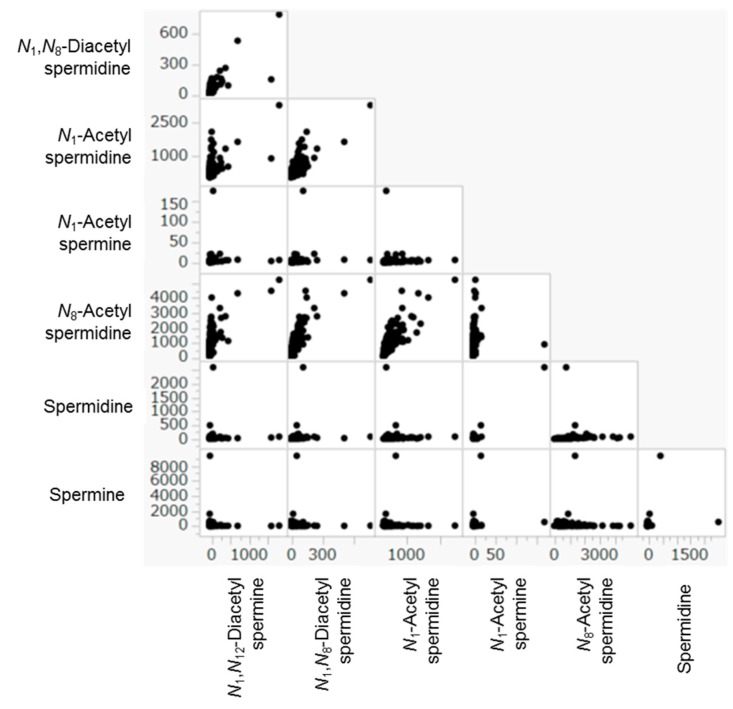
The correlation among urinary polyamines. Each dot indicates quantified data in a sample. Both the *X* and *Y* axis had no unit, since each metabolite concentration was divided by the creatine concentration. The correlation coefficients were listed at Table 4.

The correlation among urinary polyamines in M, B, and C were described using scatter plots in Figure 4 and listed at Table 4. The correlation among the models’ predictions and tumor markers in M was listed in Table 5.

## 3. Discussion

Urinary polyamines have been reported as potential biomarkers for screening CRC. However, there are concerns regarding the instability of these profiles caused by diurnal and day-to-day variation. Thus, we analyzed multiple urinary samples collected from identical individuals to confirm the large difference of polyamine concentrations between M and C, even in these variations. Subsequently, we evaluated the discrimination abilities of individual polyamines. These abilities of combinations of polyamines were also assessed using the MLR model, i.e., conventional multivariable analysis, and ADTree, one of the machine-learning techniques.

Highly positive correlations were observed among quantified metabolites in the samples corrected from M, B, and C (Figure 4 and Table 4). For example, *N*_1_-Acetylspermine showed significant correlations (*p* < 0.0001 by Spearman’s rho test) with all the other polyamines. Meanwhile, spermine showed significant correlations with only *N*_1_,*N*_12_-acetylspermidine and was independent from other polyamines.

Among quantified metabolites, *N*_1_,*N*_12_-acetylspermidine showed the highest AUC to discriminate CRC from the other groups, which was consistent with other reports [17]. The discrimination ability of *N*_1_,*N*_8_-acetylspermidine, although its accuracy was lower than *N*_1_,*N*_12_-acetylspermidine, was also observed in our data, which was also consistent with other research [17]. The elevation of polyamines, including putrescine, spermine, and spermidine and their acetylated forms, in CRC tissue and low-invasively available biofluids, such as blood and urine, while the individual metabolite alone showed little value for CRC diagnosis, indicates low specificity [6,18,19]. Therefore, we evaluated the discrimination ability of their combination.

Both mathematical models, MLR and Adtree, showed better accuracy than single metabolites alone. The accuracy of ADTree was higher than that of MLR using our data. Among various reports not limited to cancer-specific biomarker topics, various machine-learning techniques were evaluated and it was concluded that ADTree showed higher accuracy compared to the other machine-learning methods [20,21,22] and MLR [23,24]. However, even using such methods, models to discriminate B from M + C were difficult to use, yielding worse AUC values compared to models discriminating M from B + C. In fact, the polyamines were elevated the most in M, whereas no B-specific elevations were observed, which makes it difficult to establish an accurate model for B. Additionally, our data included both high-risk and low-risk adenoma in B groups. The discrimination model should be developed to discriminate one of these adenoma groups and the others. However, the number of patients of B in this study were few. For rigorous assessment of the clinical utility of our markers, more patients with both polyp groups should be involved.

The correlation among models’ predictions and tumor markers in the samples corrected from M (Table 5) showed highly positive correlations in the MLR model and both CEA (*p* = 0.0003) and CA19-9 (*p* = 0.0034) at statistically significant levels. Meanwhile, the ADTree model showed independent prediction compared to the CEA (*p* = 0.091) and CA19-9 (*p* = 0.21), which indicate that the combination of ADTree prediction and these tumor markers has potential to enhance the accuracy to discriminate CRC from the other groups. The ADTree model showed independence. However, these tumor markers were not measured in B and C in this study. The utility of combination of multiple screening tools should be evaluated.

The number of positive subjects in different stages showed different trends between tumor markers and developed models. For example, CEA showed positive values for all subjects with stage 2 or more advanced stages, while only 83% of the cases showed positive results in stage 0. Meanwhile, subjects with relatively early stages 0, 1, and 2 were detected 100%, 97.7%, and 93.5% by the ADTree model, respectively. Therefore, tumor markers and mathematical models based on polyamines are complementary and their combined use is one possible clinical application.

There are several limitations that need to be acknowledged in this study. The bootstrap analyses indicated the small difference between upper and lower 95% CI, which indicated the high generalization ability of the developed models. However, the sample sizes, especially the number of controls and polyps. were small. This affected the diurnal and day-to-day variations assessed only by control subjects. The difference of several parameters, such as age, among the given groups was the largest limitation and, therefore, rigorous validation using a large cohort data is necessary to confirm the generalization ability of the developed models. Specificity is also an important issue for screening for CRC. Elevations of urinary polyamines were reported for patients with not only CRC but also other diseases, such as breast cancer [10,17]. Partially elevated urinary polyamines for non-malignant gastrointestinal diseases would reduce the specificity for CRC diagnosis by using the individual polyamine concentration alone [6]. The current datasets did not include the patients with other cancers. Furthermore, comparison with larger cohorts, including patients with diabetes and other metabolic disorders, was also necessary to assess the specificity of the developed model. The combination of polyamines and other metabolites with highly sophisticated pattern recognition algorithms would enhance the specificity [9]. Taken together, more rigorous validation is necessary to confirm the generalization abilities of the developed models.

We utilized machine learning methods to evaluate the potential of combinations of multiple markers. MLR was also utilized here as a predictor for an identical purpose. In general, MLR suffers from the multicollinearity, e.g., the overfitting to the given problem by using variables showing highly positive correlation. Therefore, the use of only minimum independent variables is preferable to retain the predictor’s abilities, which limits the prediction accuracy of MLR. The common machine learning method, i.e., artificial neural networks, has similar problems. Therefore, even in the use of machine learning, feature selection is required to select variables considering the subsequent predictors. Here, we selected ADTree [25], boosted conventional decision trees, which we previously utilized and which are more robust against such problems [23,24,26]. However, the higher risk of overfitting should be carefully estimated for the use of machine learning. Another problem for the use of machine learning is interpretability of the developed model. MLR clearly defines the adjusted odds ratio of each selected variable, while most of the machine learning methods utilizes the variable in a black box way. Here, we employed interpretable methods while the prediction accuracy would be limited. Elevation of urinary *N*_1_,*N*_12_-acetylspermidine in CRC was frequently reported [10,11] while the change of other polyamines depends on the data [27]. Thus, the validation using a large cohort to confirm the predictive ability of each polyamine using a statistical way and the selection of appropriate variables are still necessary.

## 4. Materials and Methods

### 4.1. Study Design

This study was conducted according to the Declaration of Helsinki principles. The study protocol was approved by the Ethics Committee of Tokyo Medical University (No. 2346). Written informed consent was obtained from each subject before participating in the study. Patients with CRC included those who underwent chemotherapy. Patients with chronic metabolic diseases, such as diabetes, were also included.

The resected specimens were pathologically classified according to the 7th edition of the Union for International Cancer Control TNM Classification of Malignant Tumors [28]. The serum CEA and CA19-9 levels were measured using radioimmunoassay methods (Abbott, Chiba, Japan). The limit of detection of CEA was 0.5 ng/mL and that of CA19-9 was 2 U/mL. A high CEA level was defined as a level exceeding 5 ng/mL, and a high CA19-9 level was defined as a level exceeding 37 U/mL, according to the guidelines defined by the manufacturer of the test kit [29].

We collected 2 mL samples from the cubital vein after the diagnosis of colorectal cancer or as part of a routine investigation in healthy subjects. The tumor markers were measured with an electrochemiluminescent assay using Roche Diagnostic reagent kits and a Cobas 6000 automatic analyzer (Roche Diagnostics, Mannheim, Germany). In parallel, we performed enzyme immunoassays (EIA) and electrochemiluminescent assays (Roche Diagnostics, Mannheim, Germany) in 20 patients. The reference values were set to 5 ng/mL for CEA and 37 U/mL for CA19-9 [30].

### 4.2. Collection and Treatment of Urinary Samples

Urinary samples were collected at 7:00–8:00, 11:00–12:00, and 17:00–18:00 from identical healthy control subjects. They provided these urinary samples on three consecutive days. The urinary samples from CRC and benign cases were collected at one time between 9:00 and 16:00.

Urinary samples were collected in a 50 mL Falcon tube and stored at −80 °C prior to the metabolomic analyses. The urinary samples were divided into polyamines and creatinine concentrations. The urine (10 μL) was mixed with methanol (90 μL) containing 149.6 mM ammonium hydroxide (1% (*v*/*v*) ammonia solution) and 0.9 μM internal standards (d8-spermine, d8-spermidine, d6-*N*_1_-acetylspermidine, 1.6-diaminohexsne, d6-*N*_1_,*N*_8_-diacetylspermidine, and d6-*N*_1_,*N*_12_-diacetylspermine). After centrifugation at 20,400× *g* for 10 min at 4 °C, the whole supernatant was transferred to another tube and vacuum dried at 40 °C. The sample was reconstituted with 90% methanol (10 μL) and water (30 μL) and then vortexed and centrifuged at 20,400× *g* for 5 min at 4 °C. For the quantification of creatinine, a portion of the supernatant was diluted 5000 times by water. Diluted and undiluted samples of 1 μL were each injected into the LC/MS.

Individual metabolite concentrations quantified using the standard compounds were divided by the absolute concentration of urinary creatinine which was quantified by the methods described elsewhere [31].

### 4.3. LC Condition

The LC system used was Agilent Technologies 1290 Infinity (Agilent Technologies, Santa Clara, CA, USA) consisting of a HiP sampler, a quaternary pump, and a column compartment. Chromatographic separation was performed using an ACQUITY BEH C18 column (2.1 i.d. × 50 mm, 1.7 mm; Waters, Milford, MA, USA) at 40 °C. The mobile phase consisting of solvent A (0.1% formic acid and 1.5 mM heptafluorobutyric acid in water) and solvent B (1.5 mM HFBA in methanol) were delivered at a flow rate of 0.4 mL/min. The gradient elution is listed in Appendix A. The run time for an LC-MS analysis was 5 min, and the time for equilibration with 99% solvent A was set to be 5 min.

### 4.4. MS/MS Condition

MS detection was conducted on Agilent Technologies 6460 triple quadruple. The samples were analyzed using positive ion mode. Instrument parameters were set as follows: drying gas temperature at 275 °C, drying gas flow at 13 L/min, nebulizer at 55 psig, and Vcap at 3500. The specific MRM transition, fragmentor voltage, and collusion energy (CE) were optimized for each compound analyzed (Appendix B). Agilent MassHunter Qualitative Analysis and Quantitative QqQ Analysis software were used for data processing, including the MassHunter Optimizer and the Dynamic Multiple Reaction Monitoring Mode (DMRM) software features.

### 4.5. Data Analysis

All absolute concentrations (μmol/L) of polyamines were divided by that of creatinine (μmol/L), and, thus, subsequent analyses were conducted using the normalized values (no units).

We developed two mathematical models: model-M to discriminate malignant (M) from begin (B) and controls (C) and model-B to discriminate polyps from CRC and controls. Here, we utilized an ADTree, an improved form of the conventional if-then type decision tree [25]. This tree has been proven to be the most accurate among various popular classification methods, such as C4.5 and CART [32]. Previously we used this method and confirmed the higher accuracy [23,24].

To develop each model, the following procedures were conducted.

#### 4.5.1. Resampling

Each patient was randomly selected to generate virtual datasets under bias-controlled conditions, i.e., we used almost the same number of positive (M) and negative (P and C) subjects in the generated datasets for model M, and almost the same number of positive (P) and negative (M + C) subjects for model P. Resampling procedures were conducted with five different random values.

#### 4.5.2. Parameter Optimization

To optimize the boosting number (e.g., the number of nodes in an ADTree model), *k*-fold CV was conducted, where (1) the datasets were randomly separated into a *k*-1:1 ratio for training and validation, (2) a model was developed using training data and the prediction of the validation data, and (3) this procedure was repeated *k* times and the AUC value was calculated based on the prediction of validation datasets. Here, the boosting number was changed from 1 to 15 and cross validation procedures were repeatedly conducted using *k* = 2.

#### 4.5.3. Validation

The optimized mode was used to predict positive or negative values for each subject in original datasets. Overall, bootstrapping analyses were conducted 200 times to evaluate the variation of the predicted accuracy using multiple virtual datasets yielded by randomly selecting subjects, allowing for redundant selection. In addition, the 10-fold CV was also conducted 200 times with various random values.

### 4.6. Statistical Analysis

The discrimination ability of polyamines evaluated from the data observed in the samples collected at the morning on the first day was used for M. This is because multiple samples were collected from the patients with M.

The multiple logistic regression model was developed with backward stepwise variable selection. Variables with *p* > 0.05 were eliminated from the model. The accuracy of each model was assessed by the area under the receiver operating characteristic (ROC) curve (AUC). The Kruskal-Wallis test with Dunn’s multiple comparison was used to evaluate the difference among multiple groups. Weka data mining software (ver.3.6.13, The University of Waikato), JMP (ver. 13.2.0, SAS Institute Inc., Cary, NC, USA) and GraphPad (ver. 5.0.2 Graphpad Software, San Diego, CA, USA) were used for all analyses.

## 5. Conclusions

This study aimed to discriminate CRC from the other conditions by using urinary metabolites quantified by LC-QqQMS to profile the seven kinds of polyamines. Among all polyamines, *N*_1_,*N*_12_-diacetylspermine showed the highest differentiation ability. The area under the receiver operating characteristic curve (AUC) was 0.794 (95% CI: 0.704–0.885, *p* < 0.0001) to differentiate M from B + C. In enhancing the discrimination ability of CRC from polyps and healthy controls using combinations of polyamines, ADTree showed high AUC values, i.e., 0.961 (95% CI: 0.937–0.984, *p* < 0.0001). The methods demonstrated in this study showed the potential of CRC as a screening tool.

## Figures and Tables

**Table 1:** Subject characteristics.

Group	*n* ^1^	Age ^1^	Sex (F/M) ^2^	Risk or Location ^3^		Stage	T	N	M
C	17	42.1 ± 2.8	4/13						
B	14	65.0 ± 3.1	3/11	L	9				
				H	5				
M	201	68.7 ± 0.8	87/114	C	127	0	1	113	194
				R	74	Tis	2	49	7
						1	27	27	
						2	28	12	
						3	109		
						4a	18		
						4b	16		
*p*-Value	<0.0001	0.0912	<0.0001						

^1^ The Mann-Whitney test was used for the *p*-value. ^2^ χ^2^ test was used for the *p*-value. F and M indicated females and male, respectively. ^3^ L and H indicated high and low risk, respectively. C and R indicated colon and rectum, respectively.

**Table 2:** Multiple logistic regression (MLR) model to discriminate M from B + C and B from M + C.

	M from B + C				B from M + C			
Variables	Coefficients	95% CI		*p*-Value	Coefficients	95% CI		*p*-Value
(Intercept)	0.546	−0.357	1.45	0.24	−1.75	−2.78	−0.735	0.00080
*N*_1_,*N*_12_-diacetylspermine	7.88 × 10^4^	2.29 × 10^4^	1.35 × 10^5^	0.0057				
*N*_1_,*N*_8_-diacetylspermidine	6.73 × 10^4^	1.25 × 10^4^	1.22 × 10^5^	0.016	−6.14 × 10^4^	−1.12 × 10^5^	−2.21 × 10^4^	0.0075
*N*_1_-acetylspermidine	−9.06 × 10^3^	−1.25 × 10^4^	−5.59 × 10^3^	<0.0001	4.70 × 10^3^	1.88 × 10^3^	7.98 × 10^3^	0.0022
*N*_1_-acetylspermine	−3.09 × 10^5^	−5.71 × 10^5^	−4.63 × 10^4^	0.021				
Spermidine	5.50 × 10^4^	1.68 × 10^3^	1.08 × 10^5^	0.043				
Spermine	−5.49 × 10^3^	−9.00 × 10^3^	−1.98 × 10^3^	0.0022				

**Table 3:** Tumor markers and prediction models to discriminate M from C + B.

Marker or Model	Stage ^1^	0	1	2	3a	3b	4
	N	6	43	62	43	32	15
CEA	Av. ± SD	10.0 ± 21.7	3.29 ± 3.02	19.7 ± 51.9	6.68 ± 12.0	10.9 ± 13.1	8.92 × 10^2^ ± 2.55 × 10^3^
	>5.0 ng/mL ^3^	5 (83)	37 (86)	62 (100)	43 (100)	32 (100)	15 (100)
CE19-9	Av. ± SD	13.7 ± 20.7	15.4 ± 30.5	25.7 ± 75.1	19.5 ± 26.0	88.9 ± 1.70 × 10^2^	2.67 ± 10^3^ ± 7.12 × 10^3^
	>37 U/mL ^3^	1 (16.7)	2 (4.7)	8 (12.9)	6 (14.0)	13 (40.6)	8 (53.3)
MM ^2^	Av. ± SD	2.50 × 10^−5^ ± 3.30 × 10^−5^	2.30 × 10^−5^ ± 4.50 × 10^−5^	3.80 × 10^−5^ ± 4.60 × 10^−5^	3.00 × 10^−5^ ± 3.70 × 10^−5^	5.40 × 10^−5^ ± 9.20 × 10^−5^	3.69 × 10^−4^ ± 6.05 × 10^−4^
	>9.0 × 10 ^3^	4 (66.7)	41 (95.3)	62 (100)	42 (97.7)	31 (96.9)	14 (93.3)
MLR	Av. ± SD	0.854 ± 0.114	0.850 ± 0.214	0.950 ± 0.0618	0.938 ± 0.115	0.939 ± 0.114	0.983 ± 0.0316
	>0.81 ^3^	6 (100)	42 (97.7)	58 (93.5)	43 (100)	24 (75)	9 (60)
ADTree	Av. ± SD	0.878 ± 0.0922	0.714 ± 0.291	0.865 ± 0.144	0.809 ± 0.234	0.756 ± 0.244	0.949 ± 0.0371
	>0.722 ^3^	5 (83.3)	34 (79.1)	53 (85.5)	41 (95.3)	21 (65.6)	8 (53.3)

^1^ Av. and SD indicates average and standard deviation, respectively. ^2^ MM indicates metabolite maker; *N*_1_,*N*_12_-diacetylspermine. ^3^ The number of the subjects who showed higher than threshold levels, and parenthesized numbers show the percentage of the number of the subjects. The thresholds for MM, MLR, and ADTree had no units.

**Table 4:** Correlation of urinary polyamine concentrations ^1^.

Polyamine ^2^	7	1	2	3	4	5
1	0.763					
	(<0.0001)					
2	0.447	0.675				
	(<0.0001)	(<0.0001)				
3	0.423	0.505	0.437			
	(<0.0001)	(<0.0001)	(<0.0001)			
4	0.539	0.794	0.824	0.450		
	(<0.0001)	(<0.0001)	(<0.0001)	(<0.0001)		
5	0.455	0.620	0.586	0.465	0.785	
	(<0.0001)	(<0.0001)	(<0.0001)	(<0.0001)	(<0.0001)	
6	0.056	0.137	0.105	0.425	0.141	0.463
	(0.396)	(0.038)	(0.11)	(<0.0001)	(0.033)	(<0.0001)

^1^ Spearman’s rho and *p*-value (parenthesized value). ^2^ Numbers indicated. 1: *N*_1_,*N*_8_-Diacetylspermidine, 2: *N*_1_-Acetylspermidine, 3: *N*_1_-Acetylspermine, 4: *N*_8_-Acetylspermidine, 5: Spermidine, 6: Spermine, and 7: *N*_1_,*N*_12_-Diacetylspermine.

**Table 5:** Correlation among models’ predictions and tumor markers ^1^.

Polyamine ^2^	CEA	CA19-9	ADTree
CA19-9	0.424		
	(<0.0001)		
ADTree ^2^	0.120	0.0872	
	(0.0908)	(0.2184)	
MLR ^2^	0.255	0.206	0.165
	(0.0003)	(0.0034)	(0.0194)

^1^ Spearman’s rho and *p*-value (parenthesized value). ^2^ Predicted values of models to discriminate M from B and C.

## Abbreviations

CRC	colorectal cancer
AUC	area under the receiver operating characteristic curve
CI	confidence interval
CEA	serum carcinoembryonic antigen
ODC	ornithine decarboxylase
SSAT	spermine/spermidine *N*_1_-acetyltransferase
APAO	*N*_1_-acetylpolyamine oxidase
SMO	spermine oxidase
APC	adenomatous polyposis coli
LC-MS	liquid chromatography-mass spectrometry
CoV	coefficient of variation
MLR	multiple logistic regression
C	controls
B	benign
M	malignant
SD	standard deviation
ROC	receiver operating characteristic
ADTree	alternative decision tree
CV	cross validation

## Appendix A

**Table A1:** Liquid chromatography conditions for polyamine analysis.

Time (min)	Mobile Phase A (%)	Mobile Phase A (%)
0	99	1
0.6	99	1
0.8	58	42
1.8	58	42
2.3	50	50
3	50	50

## Appendix B

**Table A2:** MRM scans for polyamines.

Compound	Q1 ^1^ (*m*/*z*)	Q3 ^2^ (*m*/*z*)	Flag (v)	CE (v)	CAV (v)	RT (min)
1,6-diaminohexane	117.1	100.1	70	9	4	2.658
spermidine-d8	154.2	32.1	95	41	4	3.426
*N*_1_-acetylspermidine-d6	194.2	106.0	100	17	4	2.924
spermine-d8	211.3	120.1	100	21	4	3.958
*N*_1_,*N*_8_-diacetylspermidine-d6	236.2	103.1	125	17	4	2.779
*N*_1_,*N*_12_-diacetylspermine-d6	293.3	103.1	120	25	4	3.263
spermidine	146.2	72.1	95	13	4	3.426
*N*_1_-acetylspermidine	188.2	100.0	105	17	4	2.928
*N*_8_-acetylspermidine	188.2	114.0	115	17	4	3.064
spermine	203.2	112.0	100	17	4	3.958
*N*_1_,*N*_8_-diacetylspermidine	230.2	100.0	120	17	4	2.779
*N*_1_-acetylspermine	245.2	100.0	110	21	4	3.637
*N*_1_,*N*_12_-diacetylspermine	287.2	100.0	125	25	4	3.263

^1^ Preursor; ^2^ Product.
